# CHART: a novel system for detector evaluation against toxic chemical aerosols

**DOI:** 10.1038/s41598-023-50718-9

**Published:** 2024-01-10

**Authors:** Dinesh Durán Jiménez, Tom Venema, Mirjam de Bruin-Hoegée, Duurt P. W. Alkema, Ruud W. Busker, Arjan L. van Wuijckhuijse

**Affiliations:** 1 Department of CBRN Protection, TNO Defence, Safety and Security, Lange Kleiweg 137, 2288GJ Rijswijk, The Netherlands; 2 Present Address: van ‘t Hoff Institute for Molecular Sciences, Faculty of Science, University of Amsterdam, P.O. Box 94157, 1090GD Amsterdam, The Netherlands

**Keywords:** Engineering, Analytical chemistry, Chemical safety

## Abstract

Concern over the possibility of deliberate dispersion of chemical warfare agents and highly toxic pharmaceutical based agents as persistent aerosols has raised the need for experimental assessment of current and future defensive capabilities of armed forces and law enforcement agencies. Therefor we herewith present the design, realization and validation of the Chemical Hot Aerosol Research Tool (CHART) as a validated and safe experimental set-up for performance evaluation of chemical detection and identification equipment against chemical warfare agents and other highly toxic compounds. In the CHART liquid and solid compounds in solution or suspension are being dispersed as aerosols in a nebulization chamber. A broad dynamic particle size range can be generated, including particles known to be able to reach the lower respiratory tract. The aerosol generated is presented to the detection system-under-test while being monitored and characterized in real-time, using an optical particle counter and a time-of-flight aerosol analyzer, respectively. Additionally, the chemical composition of the aerosol is ex situ measured by analytical chemical methods. Evidently, in the design of the CHART significant emphasis was placed on laboratory safety and containment of toxic chemicals. The CHART presented in this paper has proven to be an indispensable experimental tool to study detectors and fieldable identification equipment against toxic chemical aerosols.

Deliberate release of chemical warfare agents (CWAs) and pharmaceutical-based agents (PBAs) is of increasing concern, both from a civil security as from a military perspective^1–3^. Both CWAs and PBAs are toxic chemical substances with a potential to injure, incapacitate or kill^4^. The primary categories of CWAs are nerve agents, blister agents, choking agents and blood agents, of which nerve agents are the most precarious^5^. PBAs include synthetic opioids, such as remifentanil and carfentanil with toxicities that even exceed those of nerve agents^6^. In a briefing guide for first responders of the US Drug Enforcement Administration accidental exposure to these aforementioned synthetic opioids by first responders is considered a “real danger”^7^. Many reports have been composed detailing developed symptoms of police officers, fire-fighters and medical service providers responding to incidents in environments where illicit drugs were present^8^. Although nerve agent exposure is less common, terrorist organizations and rogue states have deployed nerve agents on civilians and armed forces^9,10^. Whereas, based on their intrinsic toxicity, many nerve agents and PBAs are capable of causing serious injury or death, it is the dispersion method and the accuracy of its delivery that determines the actual human exposure and thereby the overall effect^11^.

Classical CWAs, including many nerve agents, typically are volatile liquids that are readily dispersed as vapors. Many of the conventional detection and identification systems are based on traditional analytical chemical analysis techniques, such as photoionization detectors, gas chromatography – mass spectrometry and ion mobility spectrometry^12^. As such, these instruments are commonly reliant on the presence of a vapor^11^. In contrast, the clandestine development of more advanced nerve agents known as the fourth generation agents or Novichock agents has led to persistent agents of very low volatility. The low to non-volatility of these compounds ensure that they can proficiently be generated as an aerosol and will remain in the aerosol phase, potentially entering the body through inhalation, dermal exposure or ingestion^12^.

The new generation of detection systems designed to detect low-volatile aerosols frequently include identification capabilities. These systems go beyond mere presence detection of a general agent class and have the capacity to identify the chemical composition of the aerosols, allowing for more precise responses and appropriate countermeasures to be taken^13^. As a consequence, these technologies rely on the substance-specific physicochemical properties of the corresponding compound for identification, such as molecular mass, ionizability or ion mobility^11^. Hence, the evaluation of detection devices has to be performed with the actual CWAs or PBAs, and aerosols of these compounds can rarely be substituted by less toxic simulants. This emphasizes the need to use the highly toxic compound itself, referred to henceforth as the “hot” agent. As a consequence, there is a corresponding need for new detector development and prototyping and the means to be able to evaluate the performance of aerosol detection equipment in a safe laboratory setting.

The goal of this study has been the development and validation of a hot agent research tool, the Chemical Hot Aerosol Research Tool (CHART), that was designed for detector evaluation of military off-the-shelf and commercially off-the-shelf detectors. The CHART was designed with requirements aimed at emulating representative scenarios, inspired by the department of defense development XM12 AVCAD program and the NATO D/100 requirements^14,15^. As an approximation a dynamic concentration range between 0.01 and 1 mg/m^3^ at a particle size distribution between 0.5 and 5 µm was chosen in this study^14,15^. A release of low-volatile CWAs, such as the nerve agent VX, typically results in an aerosol containing low volatile particles of various sizes^16^. Likewise, an aerosol consisting of solid particles deployed in a deliberate or accidental release will result in a polydisperse size distribution^17^. For an aerosol consisting of a persistent CWAs, inhalation is considered to be the primary exposure route^18^. From these polydisperse disseminations, health risk assessments estimate the thoracic fraction for adults at 50% cut-size to be at approximately 3 µm aerodynamic diameter^19^. For potent nerve agents, such as VX, even very low concentrations may cause severe and immediate health effects. The level at which VX causes irreversible health effects can vary based on factors such as exposure duration, individual susceptibility, and environmental conditions. The acute exposure guideline levels (AEGL) of the US environmental protection agency indicate that a 10-min exposure of 0.029 mg/m^3^ already may result in life-threatening health effects^20^.

The CHART offers the means to evaluate detectors and fieldable identifiers against aerosolized hot agents of known and tuneable particle size distribution of relevant concentrations in a well-defined environment. The evaluation capabilities of CHART allows measurement of aerosol characteristics in a time-resolved fashion. During a test and evaluation experiment both analytical chemical and aerosol-physical methods are applied to measure compound-specific properties. Test and evaluation of detectors against hot agents requires a prominent structure of safety measures and monitoring capabilities. In this paper the design, realization and validation of the CHART, including its test and evaluation capabilities, are provided.

## Design and operating principle

Detector evaluation against toxic chemical aerosols requires a test and evaluation facility that can simultaneously control and characterize the particle size distribution. This is achieved while measuring the challenge levels, in terms of mass per volume, both in real-time and off-line fashions for an extended amount of time to qualify detector response time and reliability. For detector evaluation mimicking a reproducible steep increase in aerosol concentration is required to enable determination of detector response time. In all cases particle size distributions should be reproducible in terms of geometric mean and standard deviation within the size range of 0.5–5 µm in order to simulate an aerosol that can form an inhalation hazard and to enable comparison of results. The particle concentration in the exposure chamber is monitored in-line in a time-resolved fashion to verify that both the desired concentration profile and the particle size distribution are generated. Offline fractions of both the vapor, solid and liquid phase need to be collected for quantitative chemical and gravimetric analysis as an orthogonal control. Vapors are of interest as some low or non-volatile chemicals might decompose during the path from the source to the detector forming volatile fragments.

### Device overview

**Figure 1:**
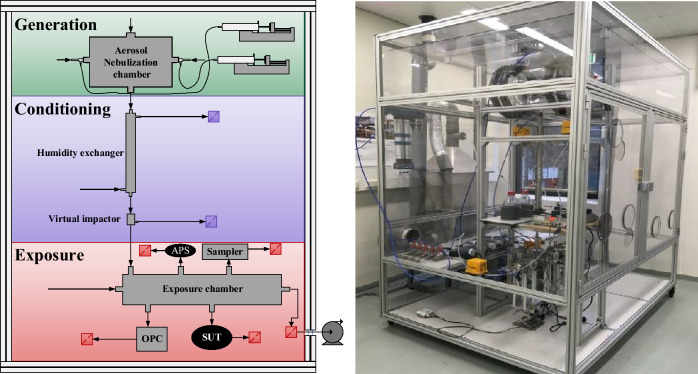
Left: The three main parts of the CHART: Generation, Conditioning and Exposure in the aluminum containment casing. Right: The CHART as realized in the laboratory. The external volume of the CHART is 7.50 m^3^.

The CHART has been designed as a multi-applicable research tool for detector evaluation against hot agents with future extensions to study physical protection, agent fate and toxicology. The CHART is designed in a modular fashion offering experimental and safety benefits to the operators using it. Individual components can be exchanged and replaced to configure different modes of operation of CHART that can have conflicting experimental conditions. Additionally, inherent toxicity and persistency of the materials dispersed in the CHART may lead to trace contaminants that cannot be removed entirely from the CHART preventing users to declare parts “clean” or “safe” without extensive validation. In the design of CHART mainly replaceable components have been used in all parts that are in direct contact with the agents, so that the complete removal of contaminants is achieved by replacing contaminated components individually for their clean counterpart. A rigorous component replacement procedure is especially needed when a next series of experiments involves a new class of agent The CHART can be divided into three main functionalities: generation, conditioning and exposure, as illustrated in Fig. 1 left, based on the functionality. In Fig. 1 right, a picture of how the CHART is realized in the laboratory is provided.

### Generation

Various commercially available options for aerosol generation have been considered. Solid aerosol generators can generate an aerosol from a solid substrate of known particle size distribution hence making the conditioning steps easier, because no drying is required, whereas liquid substrate aerosol generators may need additional drying steps^21^. The main disadvantage of a solid aerosol generator is the dependency on the size characteristics of the starting material on the aerosol that is eventually generated. Additionally, the majority of the nerve agents are viscous liquids excluding the use of a solid generator. For the CHART the PFA Meinhard liquid aerosol generator (PolyPro ST Nebulizer, Meinhard, Golden, US) was chosen to generate an aerosol from both solid and liquid substrates. Aerosols can be generated from a pure liquid, a suspension or a solution of dissolved agents that are loaded in a liquid syringe that is coupled to the Meinhard nebulizer with a microflow capillary. The Meinhard nebulizer generates a polydisperse aerosol consisting of droplets with an average mass median aerodynamic diameter (MMAD) of 12.9 µm in aqueous solution and 6.8 µm MMAD in organic solvents upon generation^22,23^. The concentration of the agent in solution can be adjusted in order to obtain an aerosol with a particle size distribution representing the thoracic fraction of a polydisperse aerosol release (i.e. between 0.5 and 5 µm). Since the volume of a spherical particle with a diameter of 2 µm is 0.4% of the volume a 12.9 µm diameter particle, a concentration of 4 mg/mL need to be applied for solution of agents with unit density in aqueous solution to yield dry particles with a MMAD of approximately 2–3 µm. The CHART offers the capability to generate aerosols from two sides in a glass cylinder shaped aerosol nebulization chamber, enabling simultaneous aerosolization of two different compounds. The aerosol nebulization chamber consists of 3 glass parts mounted together with a chemical resistant plastic sealing and has a volume of approximately 40 L. The generated aerosol is distributed in the chamber with gas flows from the Meinhard nebulizer itself and two additional mixing flows located at the top of the aerosol nebulization chamber. The additional airstream is added to reduce coagulation of the generated aerosol and to provide additional drying capacity. The generated aerosol is fed to the aerosol conditioning unit where the particle size range as well as the humidity can be adjusted.

### Conditioning

**Figure 2:**
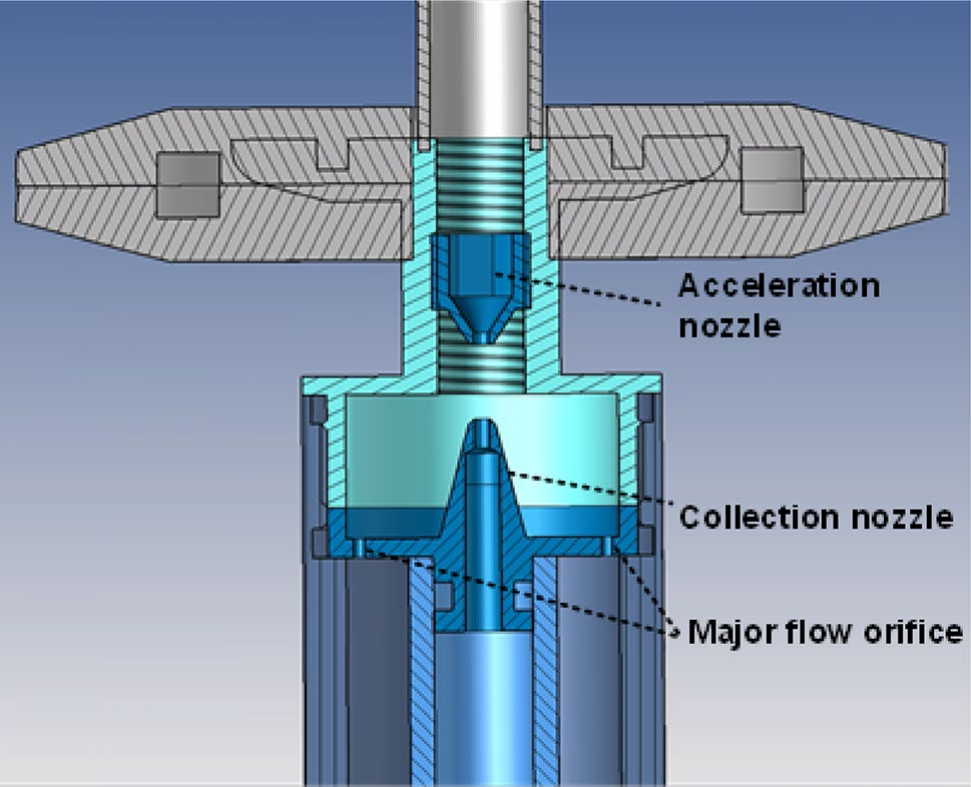
Schematic representation of the virtual impactor used in the CHART. Particles flow from the top through the acceleration nozzle with a flow of 9 L/min and are collected at the collection nozzle with a flow of 1 L/min or let through a filter via the Major flow orifice with a flow of 8 L/min.

Upon generation aerosols do not necessarily have the particle size distribution of interest, i.e. a constant particle size distribution of 0.5–5 µm. The particle size distribution and the MMAD of the aerosol can be influenced by drying or humidifying the generated aerosol. Also certain size ranges of interest can be selected for experiments in the exposure chamber. The preceding interventions are referred to as aerosol conditioning. In the CHART aerosol conditioning is achieved by employment of a 90 cm Nafion humidity exchanger (MD-700, Perma Pure, Lakewood, US) followed by particle selection using an in house designed and developed virtual impactor. The humidity exchanger installed underneath the nebulization chamber is employed to actively control the drying speed of the aerosol droplets by either enhancing solvent evaporation from the aerosol to a dry stream or from a solvent saturated gas stream to the aerosol. A Nafion humidity exchanger opposed to a silica dryer was chosen, because only solvent molecules can permeate through the Nafion tube wall, hereby creating a clear division in a clean and ‘hot’ region. Solvent is stripped from the wet stream and exchanged to the dry stream, without contaminating the clean stream with hot agents. The Nafion humidity exchanger was chosen to be able to potentially achieve both a dry (RH = 0%) and wet (RH = 90%) aerosol stream without the need to regenerate consumables for drying. However, varying the relative humidity was not validated in this study, and all work was performed under dry conditions (RH = 0.5%). The conditioned aerosol thereafter arrives at the virtual impactor, where small particles can be extracted from the aerosol increasing the fraction of coarse particles. In the virtual impactor particles are accelerated in a small orifice called the acceleration nozzle to be collected in either the collection nozzle or discarded through the major flow orifice. A schematic view of the virtual impactor is provided in Fig. 2.

As displayed in Fig. 2, the acceleration nozzle of the virtual impactor is positioned in a thread and its position can be manually adjusted with a hex key. Rotation of the acceleration nozzle will therefore increase or decrease its distance relative to the collection nozzle. Depending on the spacing between the collection nozzle and the acceleration nozzle certain particle sizes are collected more efficiently in the collection nozzle. The virtual impactor design is derived from the design of Loo et al*.* and iteratively modified to provide the desired separation characteristics^24^. The basis for this design concept lies in the impactor theory of Marple et al*.*^25^ where the cut-off characteristics are described with the Stokes number (Stk), which is given by Eq. (1), where V_0_, ρ_p_, d_p_, C_c_, η and D_0_ are gas velocity through the acceleration nozzle, particle density, particle diameter, Cunningham’s correction factor, air viscosity and the diameter of the particle acceleration nozzle, respectively.

1 $$Stk = \frac{{V_{o} \rho_{p} d_{p}^{2} C_{c} }}{{9\eta D_{o} }}$$

**Figure 3:**
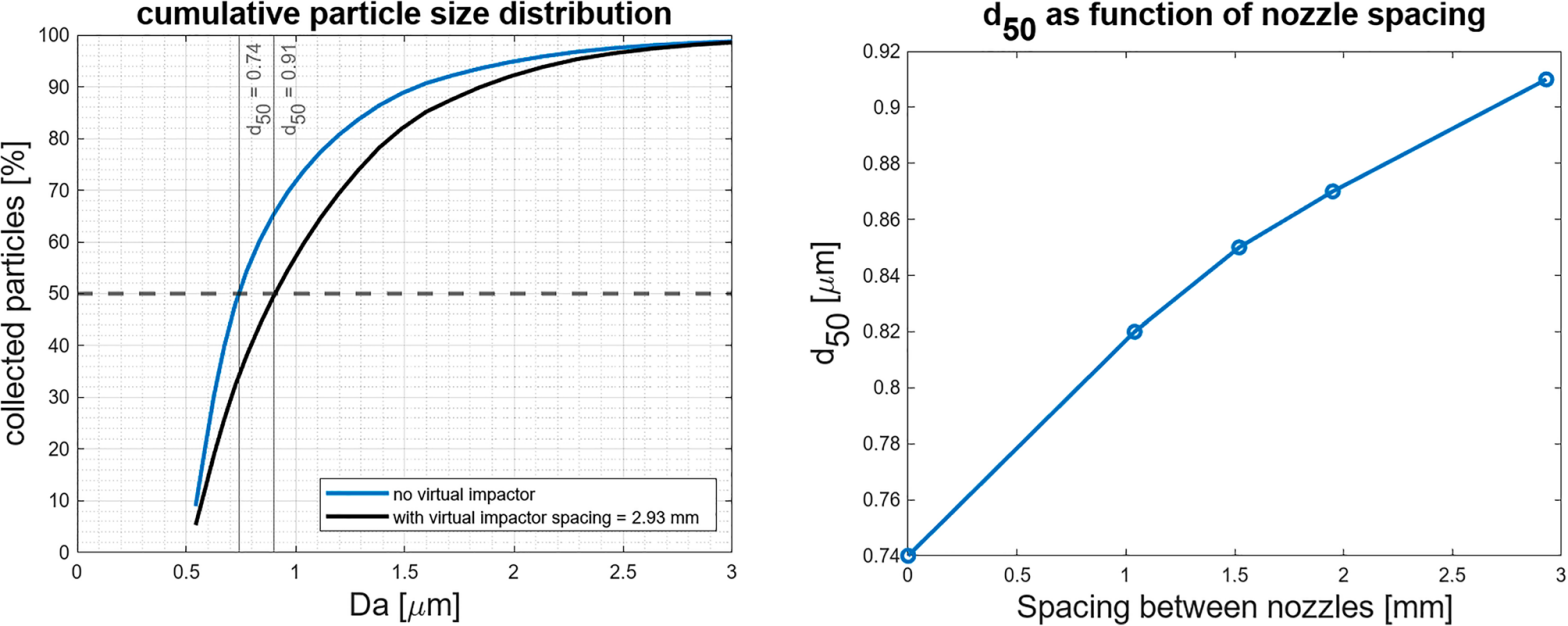
(Left) Cumulative normalized particle size distribution of TOP with and without employment of the virtual impactor with a nozzle spacing of 2.93 mm. (Right) The relation between the d_50_ and nozzle spacing.

In order to concentrate coarse particles in the collection nozzle and removing submicron particles via the major flow nozzle a Stokes number of 0.5 was used to realize a cut-off diameter of 0.5 µm. The particle laden flow of 6 L/min enters the virtual impactor acceleration nozzle from the aerosol nebulization chamber where the stream is divided over the particle collection nozzle and the major flow. The minor flow of 1 L/min leaves the virtual impactor and enters the exposure chamber, whereas the major flow of 5 L/min exits the virtual impactor through a filter out of the CHART. Tris(2-ethylhexyl) phosphate (TOP) was used to characterize the particle selection effectivity of the virtual impactor, as it possesses similar physicochemical properties of liquid organophosphorus nerve agents such as a low volatility and high viscosity, without being extremely toxic. A concentration of 0.25 mg/m^3^ was maintained in the exposure chamber for 40 min with and without a virtual impactor and with difference nozzle spacings of the virtual impactor. The cumulative normalized particle size distribution of TOP with and without employment of the virtual impactor are shown in Fig. 3 left. The application of the virtual impactor resulted in extraction of a significant part of the small particles present in the aerosol leading to a shift of the particle size distribution. The particle selection effectivity was characterized by determining the particle aerodynamic cut-off diameter (d_50_). At the d_50_ 50% of the particles of that specific size and above injected into the virtual impactor are collected in the collection nozzle. An increase in nozzle spacing, the distance between the accelerating nozzle and the collection nozzle, directly leads to an increase of the d_50_ from 0.74 to 0.91 µm due to the extraction of small particles. The particle selection effectivity as function of nozzle spacing is shown in Fig. 3 right.

The effects on the particle size distribution in the exposure chamber due to the application of the virtual impactor have been studied in more detail by monitoring the change in the statistical properties of the resultant particle size distribution that is measured with an Aerodynamic particle sizer (APS) (model APS3321, TSI, St Paul, US) in the exposure chamber. The main purpose of the virtual impactor is shifting the geometric mean of the aerosol particle size distribution while maintaining the shape. As can be seen in Table 1, a 16% increase in number based geometric mean can be achieved with a minimal increase in geometric standard deviation of 2.5%.

**Table 1:** Number based geometric mean and geometric standard deviation as function of the nozzle spacing of the virtual impactor obtained with APS.

	Geometric mean [µm]	Geometric standard deviation [-]
No virtual impactor	0.87	1.53
Nozzle spacing = 1.04 mm	0.92	1.52
Nozzle spacing = 1.52 mm	0.95	1.55
Nozzle spacing = 1.95 mm	0.98	1.57
Nozzle spacing = 2.93 mm	1.01	1.57

### Exposure

Once the desired particle size distribution is conditioned and selected, the aerosol enters the exposure chamber of the CHART. A filtered air stream is added to the aerosol if dilution is required to generate the desired concentration or to tune the velocity distribution to the sampling characteristics of the system under test (SUT). The exposure chamber consists of successive stainless steel 304 ISO-KF50 crosses with adaptors for a pressure sensor, temperature sensor, detectors, particle counters or sizers, and an aerosol/gas sampler. The CHART offers capabilities for simultaneously evaluating a SUT against a reference instruments such as an APS with or without an aerosol diluter 20:1 (aerosol diluter 3302A, TSI, St Paul, US), a Scanning mobility particle sizer (model electrostatic classifier 3082, SMPS spectrometer 3938, TSI, St Paul, US), aerosol spectrometer (Grimm 11-D, Grimm, Ainring, Germany), or an Electrical Low Pressure Impactor (ELPI) (model ELPI + , Dekati, Kangasala, Finland). Particle sizing and counting techniques such as the OPC and the APS report different “particle diameters”. Particle sizing using the OPC is based on elastic single particle light scattering according to the Mie theory, where diameters are reported in the optical diameter (d_op_). A sizing instrument such as the APS reports aerodynamic diameters (d_ae_) that are determined by the time-of-flight of particles traveling through a laser velocimeter^26,27^. Because the aerodynamic diameters are considered more relevant for studies regarding health effects and particle inhalation, optical diameters were converted to aerodynamic diameters. When the refractive index (n) of the aerosolized compound is the same as polystyrene latex (PSL) optical diameters can assumed to be equal to volume equivalent diameters and optical diameters can be converted to aerodynamic diameters following Eq. (2), shown below^26,28^.

2 $$d_{ae} = d_{op} \sqrt {\frac{{\rho_{{pC_{op} }} }}{{\rho_{0} C_{ae} \chi }}}$$

In Eq. (2), ${\rho }_{0}$ is the unit density, C_op_ and C_ae_ are the Cunningham correction factors in terms of the optical and aerodynamic diameter and $\chi$ is the dynamic shape factor. However, the refractive indices of TOP (n = 1.44) and VX (n = 1.49) used in the current study are well below that of PSL (n = 1.60). Hence, for these compounds, the empirical equation derived by Chih-Hsiang et al*.* for oleic acid (n = 1.46) was used instead to convert optical diameters to aerodynamic diameters^26^, as outlined in Eq. (3).

3 $$d_{ae} = - 0.03457d_{op}^{2} + 1.433d_{op} - 0.1157$$

In the validation of the CHART a solution of TOP in isopropanol was used to generate an aerosol. Because the used TOP is a pure liquid and the particles formed were < 1 mm in diameter a shape factor of 1 was assumed in the calculation^29^. In particle size range of interest of the CHART (0.5–5 µm) contributions from C_op_/C_ae_ are assumed to be neglectable as well. In case suspensions or solutions are used that are known to form non-spherical particles, this conversion cannot be executed as a non-unit shape factor may have drastic effects on both aerodynamic and optical equivalent diameters complicating diameter conversion, underlining the prerequisite of employing an APS in detector evaluation studies. The APS offers a well-defined particle size distribution between 0.5 and 20 micron aerodynamic diameter with 52 individual software bins and is therefore the reference of choice for detector evaluation research. The operator of the CHART selects a setpoint for a desired particle concentration in the exposure chamber and a proportional-integral (PI) control loop determines the aerosol generation of the CHART according to a pulse-width modulation (PWM) scheme directing both the liquid pump controlling the agent containing syringe and the gas flow connected to the nebulizer. A high PWM results in both a gas flow of 1 NL/min (normal liter per minute) and a liquid flow of 100 µL/min though the nebulizer whilst a low PWM results in neither a gas flow nor a liquid flow through the nebulizer. The binary behavior ensures that an aerosol with reproducible particle size distribution is generated upon activation of the Meinhard nebulizer, as a change in the nebulizer flow is known to alter the aerosol particle size distribution^22^. In the exposure chamber the conditioned aerosol is monitored with an OPC (OPC-N3, Alphasense, Essex, UK) to determine the total particle count and size distribution which is used as the process value for the PI control loop. The low cost OPC has limited aerosol characterization capabilities compared to the APS, but suffices for concentration determination for the control loop. The nebulization chamber and the exposure chamber of the CHART are spatially separated resulting in a delay of approximately 10 s between detection and particle generation. To simultaneously decrease the time required to reach the setpoint while correcting for the systems delay the Integral term was introduced in the PI controller that was optimized using the Ziegler-Nichols tuning formula^30^. The derivative term of the control loop could not be added since fluctuations in the OPC signal often yield an immense rate of change of the process value, which resulted in a uncontrollable generation.

During an exposure aerosol and gas samples can be collected, the exposure chamber is equipped with an isokinetic sampler to extract a quantified volume of aerosol. The flow is first forced through a filter and subsequently through a Tenax tube. The filter and Tenax tube combination is adopted to verify the absence of a vapor whilst proving the presence of an aerosol by capturing aerosolized compounds on a filter in the exposure chamber. Aerosolized agents could potentially decompose during the exposure, and, additionally, detection and identification systems under test may not be able to differentiate agents in aerosol or vapor phases. Therefore, it is crucial to confirm the absence or presence of any vapors to accurately assess the detection and identification capabilities for aerosolized agents. The concentration determined with the filter sampler can be used as an orthogonal control value to verify the adequacy of the aerosol characterization instruments. During the validation stage of the design no traces of agent vapor nor decomposition products were found, while typically 75% of aerosol mass was recovered from the filter, confirming the hypothesis that only an aerosol was present in the exposure chamber.

### Safety

**Figure 4:**
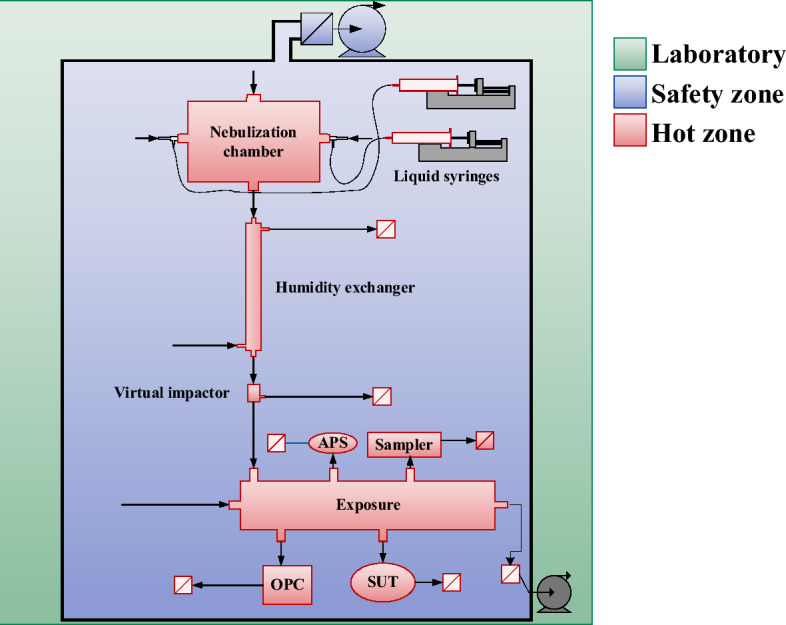
Overview of the zones in the CHART. The hot zone comprises of the space where the aerosol is generated, conditioned and exposed and is kept at a negative pressure (25 to 50 mbar below the laboratory air pressure). The safety zone consist of an extracted enclosure which houses all the auxiliary equipment that does not come in direct contact with the hot agent.

Due to the nature of the compounds used in experiments, the CHART was designed in accordance with the “safety by design” principle. The safety considerations have been integrated into the design process from the very beginning, leading to a prominent structure of safety measures. To eliminate risks of leakage of toxic agent, the CHART is divided into two zones: the hot zone and the safety zone (Fig. 4). The hot zone includes the space where the aerosol is generated, conditioned and exposed, and its boundaries form the physical barrier that separates the hot agent containing compartment from the safety zone. As mentioned earlier, individual parts confining the hot zone are regularly replaced to prevent accumulation of hot agents as an alternative to a procedure involving in situ chemical decontamination. Replaced equipment is treated with high concentrations of bleach for at least 72 h before it is disposed as chemical waste. The safety zone is the space inside the CHART casing and encloses all equipment and technique that does not require direct contact with hot agents, such as pumps, mass flow controllers and detectors. It confines a space of 6.4 m^3^ and has its own air ventilation system that streamlines an airflow into the containment zone through the admission gratings located at the bottom and side edges. The airflow is distributed evenly through the CHART components using a diffuser plate at the top and two additional exhausts at the side with a total flow of 170 m^3^/h yielding a refreshment rate of 26.5 h^−1^.

Leak tightness of the containment of the hot zone has been validated with leakage test using a trace gas detector and by using a pressure compensated flow balance. The latter is continuously monitored and logged during operation. Isopropyl alcohol was nebulized in the CHART and a leak detection test was performed using a trace gas detector (MiniRAE 3000, RAE Systems, San Jose, US). A small amount of isopropyl alcohol was nebulized in the nebulization chamber and measured from the inside using a trace gas detector as 237 ppm. The measured concentration in the safety zone fluctuated between zero and 0.1 ppm which is at the Limit of Detection (LOD) of the trace gas detector, indicating that practically no measurable amount of solvent diffuses from the hot zone into the safety zone. Accounting for the LOD of the trace gas detector this translates to a maximal concentration of 0.4 ppb which is a factor 75 above the AEGL 3 (10 min) of VX of 0.029 mg/m^3^^20^. The safety zone serves to contain agents in the case of an incident like a leakage, containment breach or obstruction. The extraction system of the safety zone was evaluated by generating a dense aerosol with a smoke generator (Vesuvius, Haagen, Amersfoort, The Netherlands). The time needed to extract the aerosol from the outer containment to the extent that no traces were visually observable was less than 15 min. This confirms that the complete volume was refreshed and no dead spaces are present in the safety zone. TOP, a persistent low-volatile simulant of organophosphorus CWAs, was dispersed in the CHART for an extended period of time and surface samples were collected at points of increased leakage risk such as joints, valves and fittings using swabs. No traces of TOP were found inside the safety zone, confirming that the nebulized agents remain inside the hot zone. All gas flows entering and leaving the system pass through HEPA/Coal filters (carbon/HEPA filter capsule, Whatman®, Maidstone, UK) that will be replaced yearly. As a passive safety measure a central pump unit continuously purges the system, maintaining a relative negative pressure of 25 mbar at the aerosol nebulization chamber and a relative negative pressure of 50 mbar at the exposure chamber with respect to the outer containment. Due to the relative negative pressure in the hot zone, potential leaks will not immediately result in a release of hot agents to the safety zone. Pressure sensors situated in the aerosol nebulization chamber and the exposure chamber monitor the aforementioned pressures.

**Figure 5:**
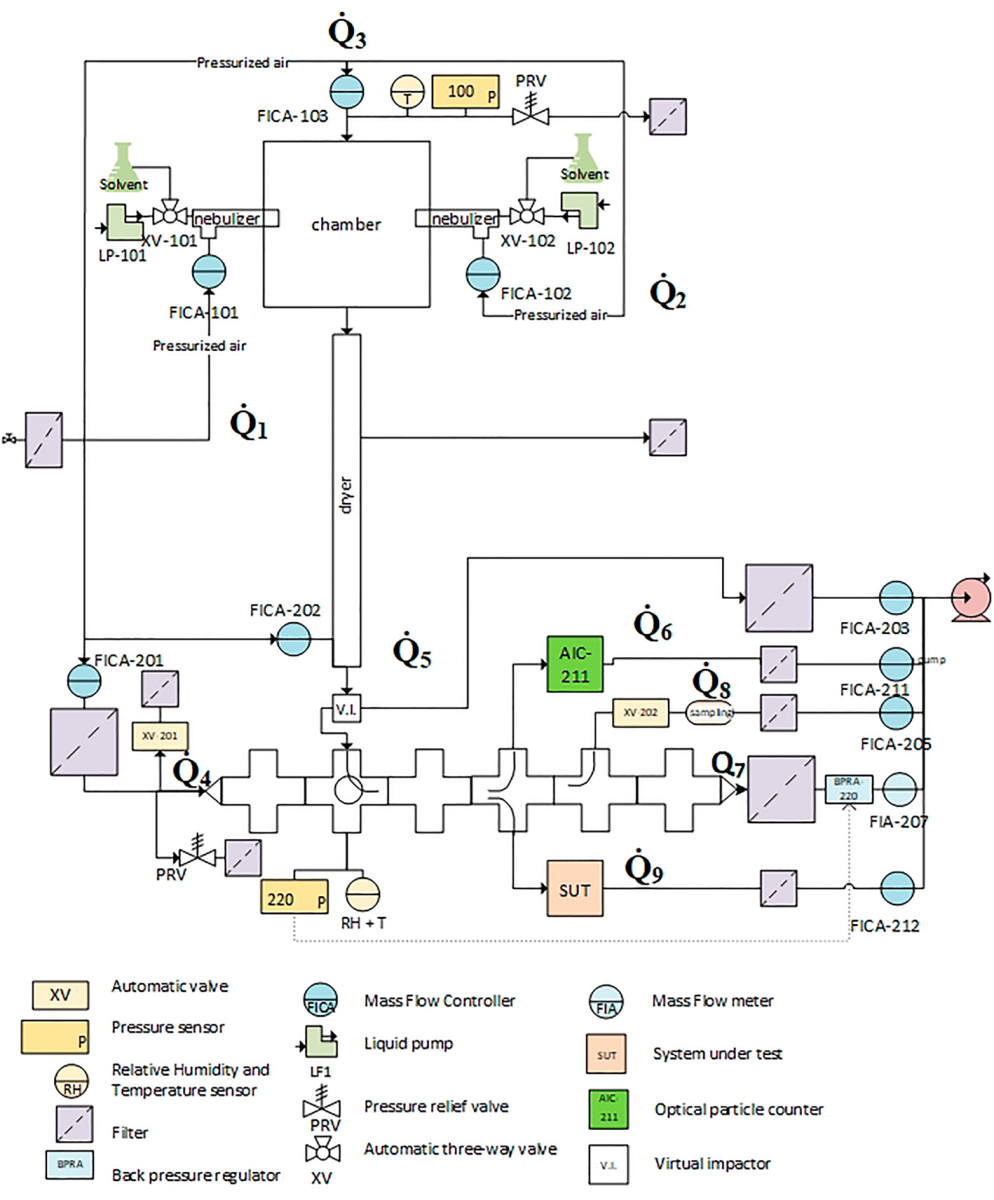
Schematic view of the components of the CHART, where the flows constituting the flow balance are indicated with Q̇.

Engineering controls will warn operators in the case of pressure or flow deviations and will automatically stop generation when a safety threshold is exceeded. Concentrations, flows, pressures and valve positions are visible to the operator at all time and can also be manipulated manually. Gas flows entering and exiting CHART are monitored with flow indicator controllers and analyzers (thermal mass flow controller F-201AV, Bronkhorst, Veenendaal, The Netherlands) with tolerances of 0.5% as can be seen in the piping and instrumentation diagram (Fig. 5).

Flows Q̇_1_, Q̇_2_ and Q̇_3_ enter the aerosol nebulization chamber and in the exposure chamber the additional dilution flow Q̇_4_ is mixed to the aerosol. The counter current flow of the humidity exchanger is not considered in the flow balance as this flow does not reach the hot agent part of CHART. Flows Q̇_5_ to Q̇_9_ exit CHART through the virtual impactor (Q̇_5_), the OPC (Q̇_6_), the main purge (Q̇_7_), sampling flow (Q̇_8_) or the system under test (Q̇_9_) respectively. When Q̇_leakage_ > 0.3 NL/min the CHART will give a warning to the operator and when Q̇_leakage_ exceeds 0.5 NL/min, CHART will automatically enter into safety modus. In safety modus aerosol generation stops by termination of the liquid pump and closure of the valves between the aerosol generator capillary and the syringe. Purging will continue until the particle concentration is zero in order to allow safe troubleshooting.

4 $${\dot{\text{Q}}}_{{{\text{leakage}}}} = {\dot{\text{Q}}}_{{1}} + {\dot{\text{Q}}}_{{2}} + {\dot{\text{Q}}}_{{3}} + {\dot{\text{Q}}}_{{4}} {-}{\dot{\text{Q}}}_{{5}} {-}{\dot{\text{Q}}}_{{6}} {-}{\dot{\text{Q}}}_{{7}} {-}{\dot{\text{Q}}}_{{8}} {-}{\dot{\text{Q}}}_{{9}}$$

In the case of a software crash a manual kill switch can be used to stop an experiment and in the case of a power blackout an uninterruptible power supply (UPS) will maintain the system long enough to finish termination mode. A battery bridges the time required for the UPS to start up and maintain the system. During this mode generation immediately stops, dilution air and the pump system remain operational, as a consequence agents present in the hot agent containment zone are diluted and flushed with clean air. The operator is also capable of flushing the hot zone with clean air manually. When particle generation ceases, the actual particle concentration decreases in accordance with the theoretical exponential decay rate of 0.15 min^-1^. As a result, the particle concentration in the exposure chamber decreases to 1% of the original concentration within 30 min.

Taking into account the equipment design and the safety evaluation performed with the smoke generator, swabs and trace gas detector in accordance with the “safety by design” principle, CHART has been demonstrated to be an intrinsic safe system for detector evaluation studies.

## Results

**Figure 6:**
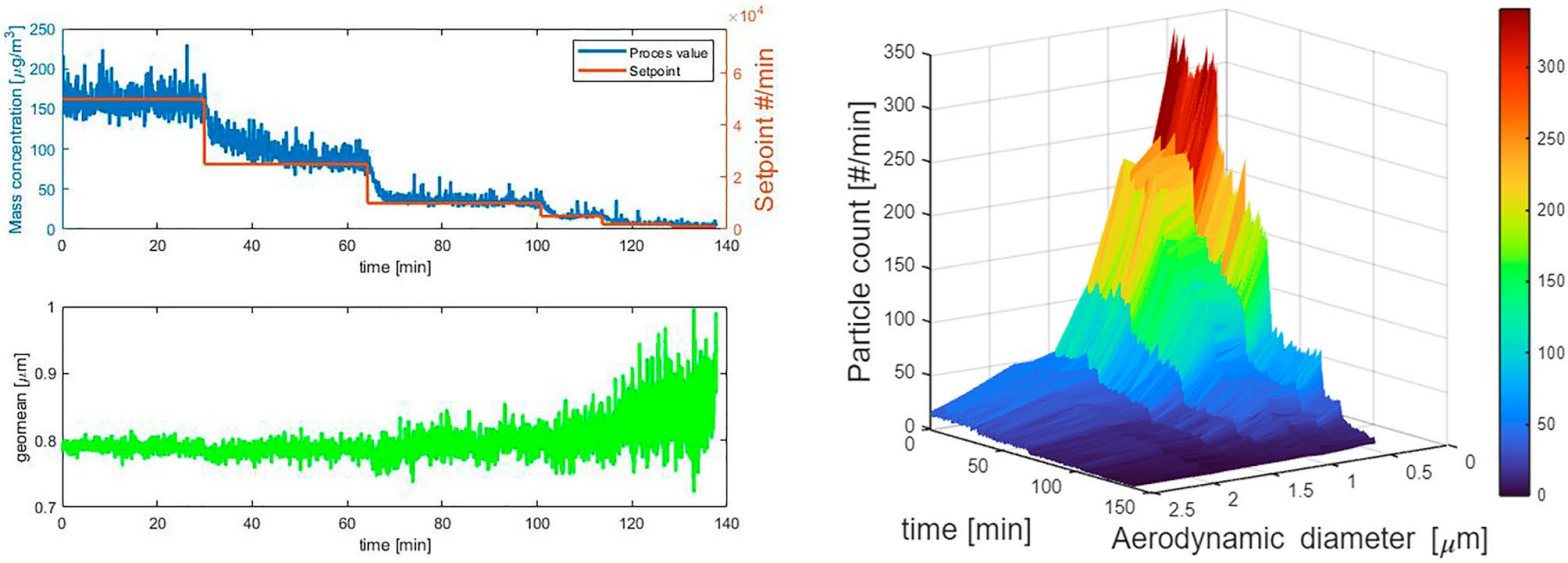
Left: Mass concentration and the moving average over 1 min in the exposure chamber of the CHART in a single dynamic run using the PI Control loop at 6 setpoints and the time resolved number particle geometric mean. Right: the corresponding particle size number distribution as function of time.

To evaluate the accuracy and stability of the PI control mechanism an exposure with the low-volatile chemical TOP was performed. Figure 6 left shows a dynamic mass concentration profile and the corresponding number geometric mean attained during these experiments. The aerosol mass concentration was adjusted several times while maintaining a constant number particle geometric mean (Fig. 6 left panel). As expected the geometric mean becomes noisier at lower particle concentrations as the particle concentration in the measurement volume of the OPC becomes so small that normalized probabilistic variation in the sample intervals increases significantly. Figure 6 (right panel) illustrates the stability of the particle size distribution in time.

Important to note is that the CHART was capable of generating an airborne mass concentration of 6 µg/m^3^, which is well below the acute exposure guideline level of relevant CWAs, such as VX (AEGL 3, 10 min, 0.029 mg/m^3^), where serious to potential life threatening effects can be expected within 10 minutes^20^.

If a detector evaluation experiment is performed, delivering a detailed particle size distribution and mass concentration measurement is required. A constant concentration of TOP was generated and measured with the APS while three particle filters and Tenax tubes for vapor samples were collected over 60 min intervals. The results of the filter sampler and the mass determination of the APS are summarized in Table 2. A concentration of 1.15 mg/m^3^ based on APS measurement was generated for 1 h in order to collect enough mass on the filter. The mass on the filter was quantified with LC–MS/MS (Supplementary Fig. S1) as an orthogonal control of the APS measurement. Blank samples were prepared by extracting clean filters that have been placed in the CHART under the same process conditions without generating particles. LC–MS/MS analysis confirmed no presence of TOP on the filters (Supplementary Fig. S2), this confirms that no re-aerosolization takes place during the operation of the CHART.

**Table 2:** Mass concentrations according to the APS and LC–MS/MS during the 60-min intervals.

Time [min]	0–60	60–120	120–180	Average
LC–MS/MS [mg/m^3^]	0.78	0.93	0.83	0.85
APS [mg/m^3^]	1.14	1.15	1.13	1.14
Recovery (%)	69	81	74	75

As can be seen from Table 2 less material was found with LC–MS/MS analysis than would be expected based on APS measurements. This can be explained with cumulative errors stemming from losses due to LC–MS/MS extraction efficiency, the APS measurement uncertainty^31^, and minor differences in gravitational and diffusional particle losses at the filter sampler and APS nozzle inlets.

**Figure 7:**
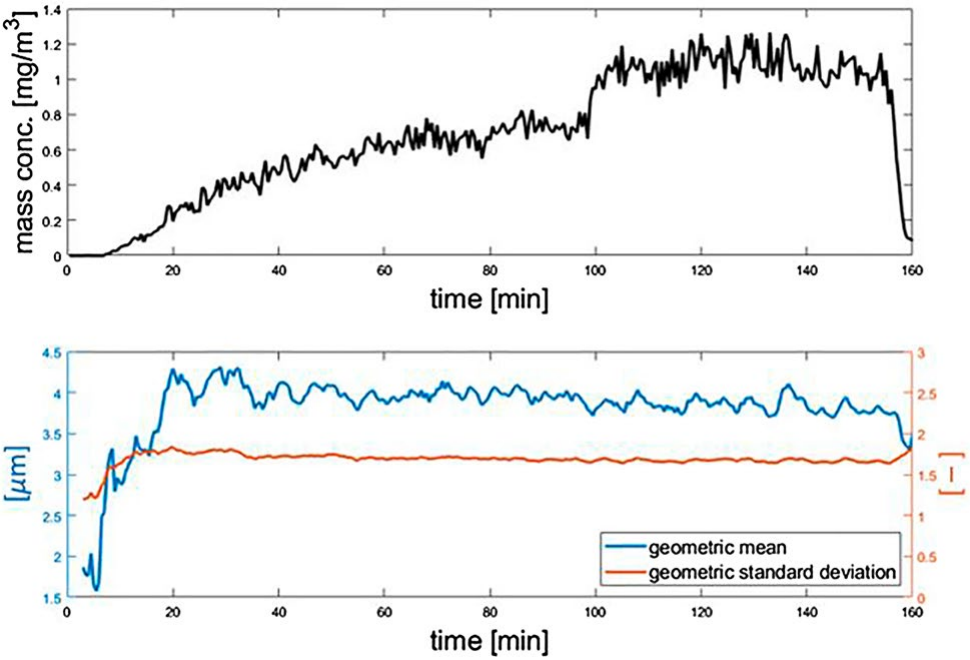
Two step concentration profile of a VX exposure for detector evaluation at 0.75 mg/m^3^ and 1 mg/m^3^ where the concentration is slowly built to the first step and rapidly increased to the second concentration.

For a detector evaluation study the nerve agent VX was dispersed in the CHART for 3 h. The VX concentration was increased once step-wise and halted after 150 min and once the dispersion stopped the aerosol mass concentration dropped sharply (Fig. 7). During the first 20 min of the exposure the particle size distribution needs to stabilize as the nebulizer and tubing still needed to flush the cleaning solvent. From t = 20 min to t = 180 the geometric mean and standard deviation remain constant regardless of the steep increase in concentration at t = 100 min. Concentration can be changed quickly to simulate representative exposure scenarios. A dynamic exposure profile consisting of a two crested wave is shown in Supplementary Fig. S3.

## Conclusion and future prospects

The CHART has been successfully designed, adopted and validated for hot agent research within a dynamic mass range of 0.01 to 1 mg/m^3^, as inspired by the XM12 AVCAD program and the NATO D/100 requirements^14,15^. The CHART has been validated for future test and evaluation applications involving detectors against hot agent aerosols in a safe and controlled environment. Aerosols are generated in the nebulization chamber and detected in situ by an APS within the 0.5 to 5 µm particle range representing the thoracic fraction, relevant for detector evaluation. A virtual impactor was shown to be capable of tuning the particle size by shifting the number geometric mean with minimal impact on the geometric standard deviation. Samples of the aerosol can be taken from the exposure chamber using filters and Tenax tubes integrated into the sampling system for ex situ GC–MS and LC–MS/MS analysis. The mass concentration sampled on the filter and evaluated with the LC–MS/MS showed an average recovery of 75% compared to the measured concentration with the APS. Filter and APS measurements confirmed the absence of any re-aerosolization processes. Analysis of the Tenax samples verified that no traces of vapors stemming from the agent or its decomposition products were present. Altogether, the results proved the applicability of CHART for detector evaluation studies. The modular design allows the CHART to also be employed for performance assessment of protective materials and toxicology research in the future. In the near future CHART will be engineered for fabric testing against highly toxic aerosols. During the current study it has been proven already that a stable particle concentrations can be generated with steep increases in concentration with a constant particle size distribution for at least 3 h. A modular part will be developed where efficiency of protective materials can be evaluated against hot agents, while taking into account the compound specific aerosol properties.

## Materials and methods

### Statement concerning safety

In this study highly toxic compounds have been used with LD_50_ values well below 1 mg/kg.

### Surface sampling

The surfaces of interest were firmly swabbed with a cotton swab. The swab was inserted in a tube with an extraction solution consisting of methanol/MilliQ (1:1 v/v) and vigorously shaken. The extraction efficiency of the swabs was estimated to be around 90%. They extraction solution was analyzed using LC–MS/MS (Waters Xevo TQ-S, with a Waters Acquity M-Class LC module, Waters, Milford, US).

### Filter extraction

A flow of 0.4 NL/min was diverted from the exposure chamber and forced through a polyether sulfone filter (0.2 µm pore size) The filters were removed from the filter sampler and extracted with 4 mL methanol/MilliQ (1:1 v/v) solution using a vortex. The extraction efficiency of the filters was estimated at 94% The concentration of the extraction solution was determined using a LC–MS/MS (Waters Xevo TQ-S, with a Waters Acquity M-Class LC module, Milford, US).

### Vapor analysis

A flow of 0.4 NL/min was diverted from the exposure chamber and forced through a Tenax TA 60/80 tube. The Tenax tubes were thermally desorbed and analyzed using a multimode inlet (OPTIC-4, GL Sciences, Eindhoven, The Netherlands) coupled to a GC–MS (an Agilent Technologies 7890A GC-system in combination with an Agilent Technologies 5975 inert XL MSD, Santa Clara, US). The injector was flash heated from 10 to 250 °C at a flow rate of 8 mL He/min. After a desorbing time of 90 s the column flow was reduced to 1.5 mL/min.

### TOP dispersion

A disposable polypropylene syringe was filled with a stock solution of 17 mg/mL TOP in IPA and was placed in the syringe pump (KDS Legato, KD Scientific, Holliston, US) in the CHART. A set point in total particle count was manually chosen via the CHART’s software interface and the particle concentration was regulated automatically via the PI control mechanism.

### VX dispersion

A disposable polypropylene syringe was filled with a stock solution of 20 mg/mL VX in IPA and was placed in the syringe pump (KDS Legato, KD Scientific, Holliston, US) in the CHART. A set point in total particle count was manually selected via the CHART’s software interface and the particle concentration was regulated automatically via the PI control mechanism.

### Supplementary Information

Supplementary Information.

## Data Availability

The datasets generated during and/or analyzed during the current study are not publicly available due to lab export control policies but are available from the corresponding author on reasonable request.
