# Supplementary material for: CHART: a novel system for detector evaluation against toxic chemical aerosols
Source: Sci Rep. 2024 Jan 10;14:1050. doi: 10.1038/s41598-023-50718-9 (PMC10781669; doi:10.1038/s41598-023-50718-9)
Supplement: Supplementary file 1 — Supplementary Information. [file 41598_2023_50718_MOESM1_ESM.pdf]

Supplementary Information

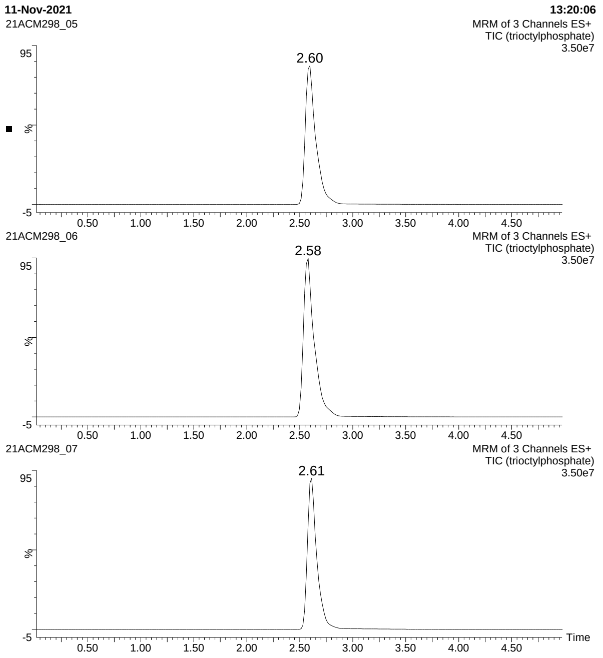

Supplementary figure S1: Chromatogram of samples 1 to 3, sum of the three transition ions.

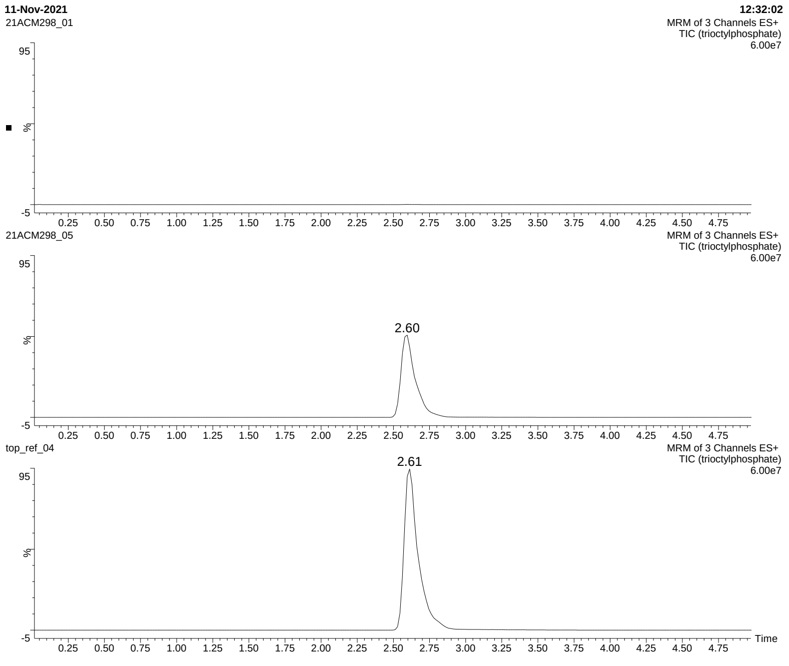

Supplementary figure S2: LC-MS/MS chromatogram of the blank, sample and reference, sum of three transition ions.

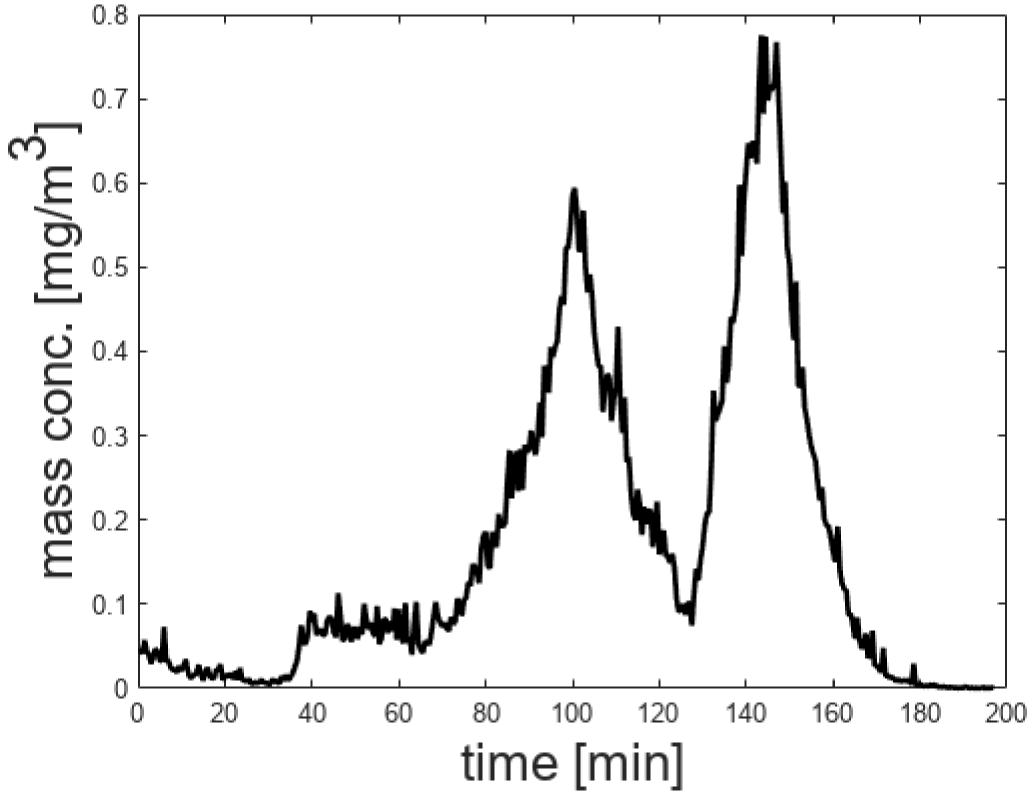

Supplementary figure S3: Dynamic exposure profile of VX consisting of two concentration crests.
